# Rationale and design of the randomized multicentre His Optimized Pacing Evaluated for Heart Failure (HOPE‐HF) trial

**DOI:** 10.1002/ehf2.12315

**Published:** 2018-07-09

**Authors:** Daniel Keene, Ahran Arnold, Matthew J. Shun‐Shin, James P. Howard, SM Afzal Sohaib, Philip Moore, Mark Tanner, Norman Quereshi, Amal Muthumala, Badrinathan Chandresekeran, Paul Foley, Francisco Leyva, Shaumik Adhya, Emanuela Falaschetti, Hilda Tsang, Pugal Vijayaraman, John G.F. Cleland, Berthold Stegemann, Darrel P. Francis, Zachary I. Whinnett

**Affiliations:** ^1^ Imperial College London London UK; ^2^ Imperial College Healthcare NHS Trust London UK; ^3^ West Hertfordshire Hospitals NHS Trust Hertfordshire UK; ^4^ Barts Health NHS Trust London UK; ^5^ West Sussex Hospitals NHS Trust West Sussex UK; ^6^ Wycombe General Hospital High Wycombe UK; ^7^ North Middlesex University Hospital London UK; ^8^ Great Western Hospitals NHS Foundation Trust Swindon UK; ^9^ University Hospitals Birmingham Birmingham UK; ^10^ Medway NHS Foundation Trust Kent UK; ^11^ Imperial College Trials Unit, Imperial College London London UK; ^12^ Geisinger Commonwealth School of Medicine Geisinger Heart Institute Scranton PA USA; ^13^ Bakken Research Center B.V. Research and Technology Maastricht The Netherlands

**Keywords:** Heart failure, His‐bundle pacing, Atrioventricular delay, Optimization

## Abstract

**Aims:**

In patients with heart failure and a pathologically prolonged PR interval, left ventricular (LV) filling can be improved by shortening atrioventricular delay using His‐bundle pacing. His‐bundle pacing delivers physiological ventricular activation and has been shown to improve acute haemodynamic function in this group of patients.

In the HOPE‐HF (His Optimized Pacing Evaluated for Heart Failure) trial, we are investigating whether these acute haemodynamic improvements translate into improvements in exercise capacity and heart failure symptoms.

**Methods and results:**

This multicentre, double‐blind, randomized, crossover study aims to randomize 160 patients with PR prolongation (≥200 ms), LV impairment (EF ≤ 40%), and either narrow QRS (≤140 ms) or right bundle branch block. All patients receive a cardiac device with leads positioned in the right atrium and the His bundle. Eligible patients also receive a defibrillator lead. Those not eligible for implantable cardioverter defibrillator have a backup pacing lead positioned in an LV branch of the coronary sinus. Patients are allocated in random order to 6 months of (i) haemodynamically optimized dual chamber His‐bundle pacing and (ii) backup pacing only, using the non‐His ventricular lead.

The primary endpoint is change in exercise capacity assessed by peak oxygen uptake. Secondary endpoints include change in ejection fraction, quality of life scores, B‐type natriuretic peptide, daily patient activity levels, and safety and feasibility assessments of His‐bundle pacing.

**Conclusions:**

Hope‐HF aims to determine whether correcting PR prolongation in patients with heart failure and narrow QRS or right bundle branch block using haemodynamically optimized dual chamber His‐bundle pacing improves exercise capacity and symptoms. We aim to complete recruitment by the end of 2018 and report in 2020.

## Introduction

In patients with heart failure and a reduced ejection fraction, a prolonged atrioventricular (AV) delay is associated with increased mortality.^1^, ^2^, ^3^ Pacing is able to reduce a prolonged AV delay; however, in patients with a narrow QRS, both right ventricular and biventricular pacing can induce additional ventricular dyssynchrony.^4^ His‐bundle pacing however avoids this as it activates the ventricles using the intrinsic conduction system.

### Prolonged atrioventricular delay is pathological in heart failure

Observational studies have found an association between a prolonged PR interval and worse outcomes.^5^ In ischaemic heart disease, patients with a long PR interval were found to have a 58% higher mortality.^5^, ^6^ In patients with heart failure, PR prolongation also has an important prognostic impact. For example, in the control arm of MADIT‐CRT (Multicenter Automatic Defibrillator Implantation Trial with Cardiac Resynchronization Therapy), those patients with a prolonged PR interval had a three‐fold higher risk of death or heart failure than those whose AV delay was within normal limits.^1^

### Prolonged atrioventricular delay can be a target

The concept of providing AV resynchronisation as a therapeutic target in heart failure predates that of ventricular resynchronisation.^7^, ^8^ Suggestions that a prolonged AV delay in heart failure is not only a prognostic marker but also a therapeutic target comes from both acute haemodynamic studies and an association with benefit found within randomized trials.

Shortening pathologically prolonged PR intervals has the potential to improve cardiac function. PR prolongation impairs LV filling,^9^ while reducing prolonged PR intervals improves LV filling.^10^ In patients with left bundle branch block (LBBB), optimizing left ventricular filling by shortening AV delay using biventricular pacing acutely improves acute cardiac function, as reflected by changes in LV dP/dt_max_, stroke volume, coronary artery flow, and systolic blood pressure (SBP).^11^, ^12^, ^13^

Furthermore, data from large randomized trials of biventricular pacing in patients with LBBB have found that patients with prolonged native PR intervals appear to gain greater benefit from biventricular pacing.^14^ In COMPANION, patients with a long PR interval had a 17% greater relative risk reduction in heart failure admissions and death compared with those with a normal PR interval. Similarly, in the MADIT‐CRT trial, the prognostic benefits of biventricular pacing appeared somewhat greater for participants with a wide QRS of non‐LBBB morphology when the PR interval was prolonged.^2^

### Right ventricular and biventricular pacing cause ventricular dyssynchrony in patients with narrow QRS

Both right ventricular pacing and biventricular pacing can be used to treat a prolonged PR interval. However, both have potential disadvantages.

### Right ventricular pacing

Conventional right ventricular pacing had been used to shorten prolonged PR intervals in small studies with mixed results.^7^, ^15^ However, conventional right ventricular pacing prolongs left ventricular activation and therefore causes dyssynchronous ventricular activation, which might offset the beneficial effects of shortening PR interval. It is therefore unlikely to be the most efficient method for delivering AV delay optimization in patients with heart failure.

### Biventricular pacing

Biventricular pacing might be a better option^16^ for delivering AV delay optimization. Biventricular pacing has transformed the care of patients with reduced left ventricular ejection fraction heart failure and a prolonged QRS duration.^17^, ^18^

The effects of biventricular pacing in patients without QRS prolongation have also been explored,^19^, ^20^ particularly focusing on those with echocardiographic mechanical dyssynchrony, because of the belief that biventricular pacing delivers its benefit mainly through resynchronization of ventricular wall contraction. However, when tested in a bias‐resistant manner, the results have not been favourable. Biventricular pacing has been found to be harmful (the most prominent trial showing an 81% increase in mortality) when applied to this group of patients.^20^

One potential mechanism for harm is that when the native QRS is narrow, biventricular pacing prolongs ventricular activation time and therefore increases ventricular dyssynchrony.^4^

Therefore, even biventricular pacing is an imperfect way to deliver AV delay shortening, because some of the beneficial effects may be offset by the non‐physiological ventricular activation.

### His‐bundle pacing

**Figure 1:**
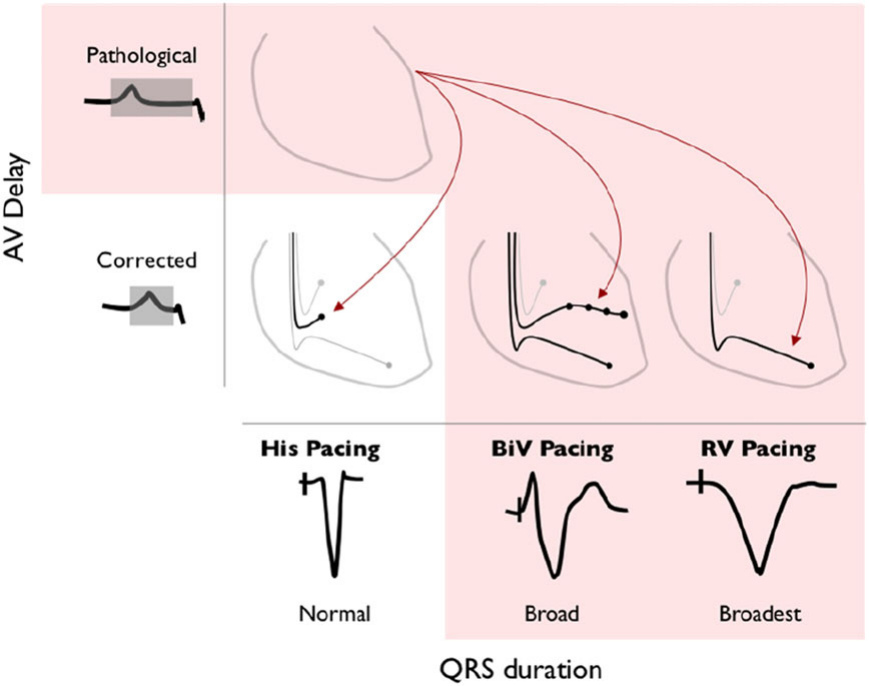
Potential pacing methods for atrioventricular (AV) delay optimization. His‐bundle pacing, biventricular (BiV) pacing, and right ventricular (RV) pacing are all potential methods for shortening a pathologically long AV delay. His‐bundle pacing does not prolong left ventricular activation time, when applied to patients with a narrow QRS duration, whereas BiV pacing and RV pacing prolong left ventricular activation time.

His‐bundle pacing may be a better way to deliver AV delay optimization in this group of patients. Permanent His‐bundle pacing allows the ventricles to be activated via the His‐Purkinje system. As a result, normal physiological cardiac activation is maintained when His‐bundle pacing is applied to people with a narrow QRS duration.^21^ It may even restore normal physiological activation in patients with right bundle branch block (RBBB) or LBBB.^22^ Chronic His‐bundle pacing has been shown to be feasible and safe when used for bradycardia pacing (*Figure* *1*).^23^

### Evidence that His‐bundle pacing may be beneficial in heart failure with prolonged atrioventricular delay and narrow QRS

In patients with heart failure, a prolonged PR interval, and either a narrow QRS or RBBB, AV delay optimization delivered using His‐bundle pacing has been shown to improve acute haemodynamic function.^24^ While this is encouraging, longer term outcome data are required before His pacing can be routinely recommended as a method for pacing therapy for heart failure in a group of patients who are not currently targeted for treatment with biventricular pacing.

There is a precedent that haemodynamic changes translate into better patient outcomes. The earliest evidence for benefit from biventricular pacing in patients with heart failure with LBBB was the clear and immediate improvement in haemodynamics seen when pacing was initiated.^8^, ^25^ Medium term physiological studies subsequently demonstrated a sustained effect with improved exercise capacity compared with controls at up to 1 year.^26^ In turn, longer term trials showed reduction in morbidity and mortality.^17^

### Aim

The aim of HOPE‐HF (His Optimized Pacing Evaluated for Heart Failure) trial is to test whether the acute haemodynamic effects observed with AV optimized His‐bundle pacing in patients with heart failure, a long intrinsic PR interval, and narrow QRS or RBBB translate into an improvement in exercise capacity, symptoms, and cardiac function.

## Methods

### Trial purpose

The HOPE‐HF trial is designed to evaluate the effects of AV optimized His‐bundle pacing for patients with left ventricular systolic dysfunction and PR prolongation but without LBBB. The primary endpoint is the within‐patient difference in exercise capacity with His optimized pacing compared with no pacing in a crossover trial with 6 month treatment periods. Secondary endpoints are quality of life (QOL), echocardiographic measurements, B‐type natriuretic protein (BNP) measurements, and daily patient activity levels.

### Trial design

HOPE‐HF is an investigator‐initiated, UK‐based, multicentre, blinded, randomized, crossover trial of permanent AV optimized His‐bundle pacing. The planned recruitment is for 160 randomized patients across approximately 20 sites in the UK.

### Inclusion and exclusion criteria

Study inclusion and exclusion criteria are shown in *Table* 1.

**Table 1:** Inclusion and exclusion criteria (EF is left ventricular ejection fraction)

Inclusion criteria
Age over 18 years
EF ≤ 35% or EF 36–40% with BNP > 250 pg/mL
NYHA class II–IV
Sinus rhythm
PR interval ≥ 200 ms
QRS duration ≤ 140 ms or prolonged QRS duration with typical RBBB morphology
Exclusion criteria
Permanent or persistent AF
Paroxysmal AF with history of sustained AF (≥24 h) in the 6 months prior to screening
Patients who are unable to perform cardiopulmonary exercise testing
Lack of capacity to consent
Other serious medical condition with life expectancy of <1 year
Pregnancy

AF, atrial fibrillation; NYHA, New York Heart Association; RBBB, right bundle branch block.

In brief, patients with symptomatic systolic heart failure with an ejection fraction ≤ 40%, a PR interval ≥ 200 ms, and either narrow QRS (≤140 ms) or RBBB of any duration are eligible. The 140 ms QRS threshold arises from a meta‐regression of individual patient data from 3782 patients in five trials.^17^ The evidence for biventricular pacing in patients with a QRS duration <140 ms is less consistent.^17^

An additional requirement for patients with a left ventricular ejection fraction between 36 and 40% is that they must have a documented plasma BNP of >250 pg/mL within the preceding 1 year.

Patients with permanent or persistent atrial fibrillation are excluded because there is no opportunity for AV delay optimization. Those with paroxysmal atrial fibrillation (with sustained episodes lasting >24 h) are only eligible once treatment to maintain sinus rhythm has been initiated and they have remained in sinus rhythm for 6 months.

### Intervention

#### Device implantation procedure

Permanent His‐bundle pacing is attempted in all patients. The trial is a multicentre study with device implantations occurring at a number of different hospitals within the UK. Implantation procedures are carried out by experienced device implanters who as part of the trial were offered training in His‐bundle pacing.

**Figure 2:**
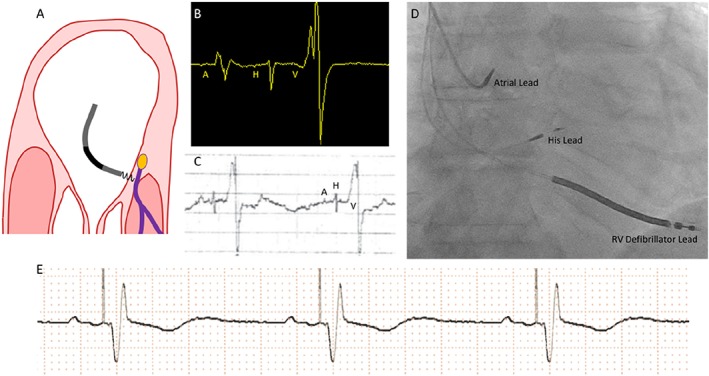
(A) Intended area within right atrium for implantation of the His pacing lead. The target area is where the non‐branching part of the His bundle sits above the tricuspid valve on the right atrial—left ventricular part of the membranous septum. (B) Appearance of a single cardiac cycle recorded on an EP system, from a well‐positioned His lead. Note that the atrial component (A) is less than half the size of the ventricular component (V). There is a His signal (H) between the two. This appearance suggests a favourable location. (C) The electrogram can also be displayed using the Pace–Sense Analyser. It is advisable to measure initially a unipolar signal utilizing the atrial channel with a gain setting of 0.05 mV/mm and a sweep speed of 50–100 mm/s. The ventricular channel is not sufficiently sensitive to detect His potentials. (D) This anterior–posterior projection radiographic image shows the typical anatomical location for the His lead (labelled). (E) Rhythm strip showing successful His‐bundle pacing. RV, right ventricular.

His‐bundle pacing is achieved using the active fixation Medtronic Select Secure 3830 lead (Medtronic Inc., Minneapolis, MN, USA). The lead is delivered using either the Medtronic C315 dedicated His delivery sheath or the Medtronic C304 deflectable sheath (Medtronic Inc.). During the implant procedure, a 12 lead electrocardiogram (ECG) is continuously recorded to allow QRS duration and morphology to be assessed. The electrogram from the His pacing lead is displayed using either an electrophysiology monitoring system or a Pace–Sense Analyser. The signal from the lead is used to locate the His bundle. We target a clear His potential followed by a ventricular signal, which is at least double the size of the preceding atrial signal (*Figure* *2*).

If His‐bundle pacing is unsuccessful, then a left ventricular lead is implanted. The third lead is either a right ventricular defibrillator lead, for patients with an implantable cardioverter defibrillator (ICD) indication, or a left ventricular coronary sinus lead, for those without an ICD indication. The left ventricular lead was suggested by the funding review committee so that a backup pacing lead is available should it become clinically required and the His pacing lead becomes non‐functional or to provide backup pacing when the His lead is programmed off (when in the non‐treatment arm). All patients receive a right atrial lead.

The generators used in the HOPE‐HF trial are conventional cardiac resynchronization therapy (CRT) pacemaker (CRT‐P) or CRT defibrillator devices.

#### Ventricular activation with His‐bundle pacing

During His pacing, the theoretical physiological ideal is selective capture of the His‐Purkinje system, so that ventricular activation occurs purely via the native conduction system and the resulting QRS is no wider than the native QRS with identical morphology. In patients with RBBB, His pacing may shorten QRS duration due to recruitment of the right bundle branch fascicles.

**Figure 3:**
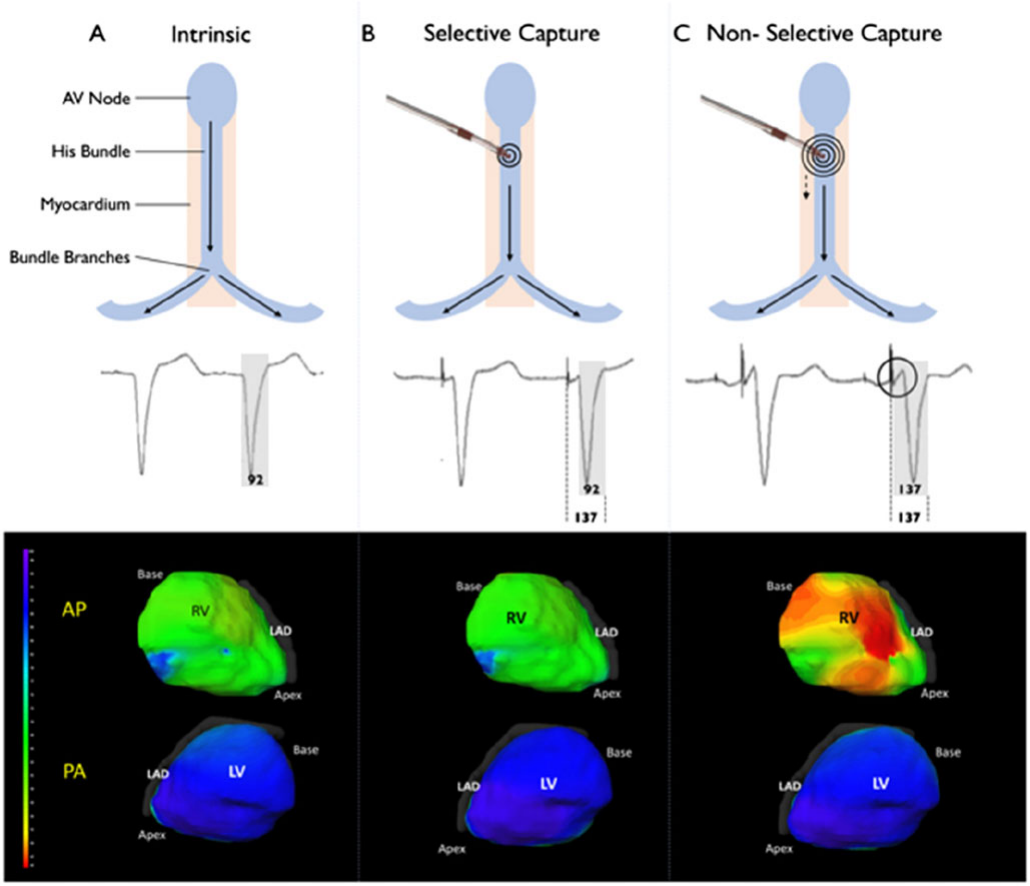
Differences between selective and non‐selective His pacing. (A) Normal intrinsic conduction. The electrocardiographic imaging (Medtronic Inc., Minneapolis, MN, USA) activation map shows rapid activation (each ventricle has homogenous colouring). (B) Selective His‐bundle pacing. Ventricular activation occurs solely via the His‐Purkinje system, giving a normal QRS and normal activation map. The middle panel shows the short delay between the pacing spike and the onset of ventricular activation. (C) Non‐selective His‐bundle pacing, during which ventricular activation occurs via both local myocardial capture [seen as early activation in right ventricle (RV) (red colour)] and activation via the His‐Purkinje system. Left ventricular (LV) activation time and pattern is the same as during intrinsic activation and with selective His pacing because activation of the LV occurs via the His‐Purkinje system. AP, anterior‐posterior orientation; LAD, left anterior descending; PA, posterior‐anterior orientation.

Non‐selective His‐bundle capture occurs when there is capture of both the His bundle and the surrounding local right ventricular myocardium close to the lead tip. Local ventricular activation occurs while the His wave front is travelling through the His‐Purkinje system (prior to myocardial breakout). This results in a pseudo‐delta wave on the surface ECG. The majority of ventricular activation occurs rapidly via the His‐Purkinje system. Overall, QRS duration is prolonged slightly during non‐selective capture, as a result of the early local myocardial activation; however, the stimulation to end QRS time is not prolonged compared with the His to end of QRS time, and reassuringly, left ventricular activation time and pattern are not prolonged with non‐selective His capture.^27^ In the HOPE‐HF trial, the aim is to achieve His‐bundle capture without prolongation of left ventricular activation time. Therefore, we accept either selective or non‐selective capture. His capture is confirmed using previously documented criteria^28^ and summarized in Supporting Information, *Table* *S1* and illustrated in *Figure* *3*.

### Study endpoints

#### Primary endpoint

The primary endpoint of the trial is peak oxygen uptake measured during cardiopulmonary exercise testing. This endpoint was selected because it is reproducible,^29^ a well‐recognized measure of cardiac function reserve, and a strong predictor of mortality in heart failure.^30^ It has been used in many previous trials of heart failure.

#### Secondary endpoints

Secondary endpoints include echo measurements of LV volume, plasma concentration of BNP, QOL scores, daily patient activity levels, and hospitalization episodes.

### Endpoint measurements

#### Cardiopulmonary exercise testing

Patients exercise on a treadmill using a smoothed modified Bruce protocol.^29^ Spirometry and gas exchange are monitored using the COSMED Quark CPX System (Cosmed, Italy). Exercise capacity is measured as peak oxygen consumption (peak VO_2_) in mL/kg/min. Exercise time, VE/VCO_2_ slope, and oxygen uptake efficiency slope are also measured.

#### Echocardiogram

Echocardiographic measurements are made using a Phillips iE33 (Phillips, The Netherlands). The following variables are measured: left ventricular dimensions, volumes and ejection fraction, tissue‐Doppler imaging, quantification of mitral regurgitation, and maximum velocity through the aortic valve. Pulsed‐wave Doppler through the mitral valve is also recorded for offline analysis of E‐A waves for assessment of AV filling patterns.

#### Quality of life

Quality of life is scored using both the Minnesota Living with Heart Failure Questionnaire and the EQ‐5D. The Minnesota Living with Heart Failure Questionnaire is a validated condition‐specific measure with good sensitivity and discrimination. It is complemented by the EQ‐5D, which is a validated generic measure of QOL, which enables comparison of findings with other outcome studies. These are completed by the patient. New York Heart Association assessment is made by trial physicians.

#### Blood samples

Blood samples are taken for analysis of plasma BNP (Abbott ARCHITECT immunoassay BNP, Abbott Laboratories, Abbott Park, IL, USA).

#### Daily patient activity levels

Daily patient activity levels are automatically recorded by the CRT device using an accelerometer. This gives a continuous assessment of daily activity levels for the entire duration of the study and has been shown to predict adverse cardiovascular events.^31^

#### Implant data

Skin‐to‐skin time for device implantation is being recorded as is total fluoroscopy duration. The delivery sheath utilized to position the His lead is being noted, and lead measurements including thresholds and sensing data for all leads are recorded.

#### Follow‐up location

All endpoint assessments and haemodynamic optimizations (to determine the patient's optimal paced and sensed AV delays) are performed at the core centre (Imperial College London). This is to ensure that investigations are carried out within the required time frame of the study, measurement variability is minimized by using the same equipment and staff, and set‐up and training costs are minimized.

#### Follow‐up duration

The study endpoint measurements are conducted at three set time points. Baseline measures are taken 2 months after the device has been implanted (during which time no His pacing is performed). The duration of each treatment arm is 6 months, to allow time for cardiac remodelling and to minimize any carryover effect. Further endpoint assessments take place 6 months after the baseline visit while patients have had either His pacing programmed on or off and then after a final 6 month interval following crossover to the alternate arm. Assessment of adverse events is continuous throughout the trial.

#### Patient flow

**Figure 4:**
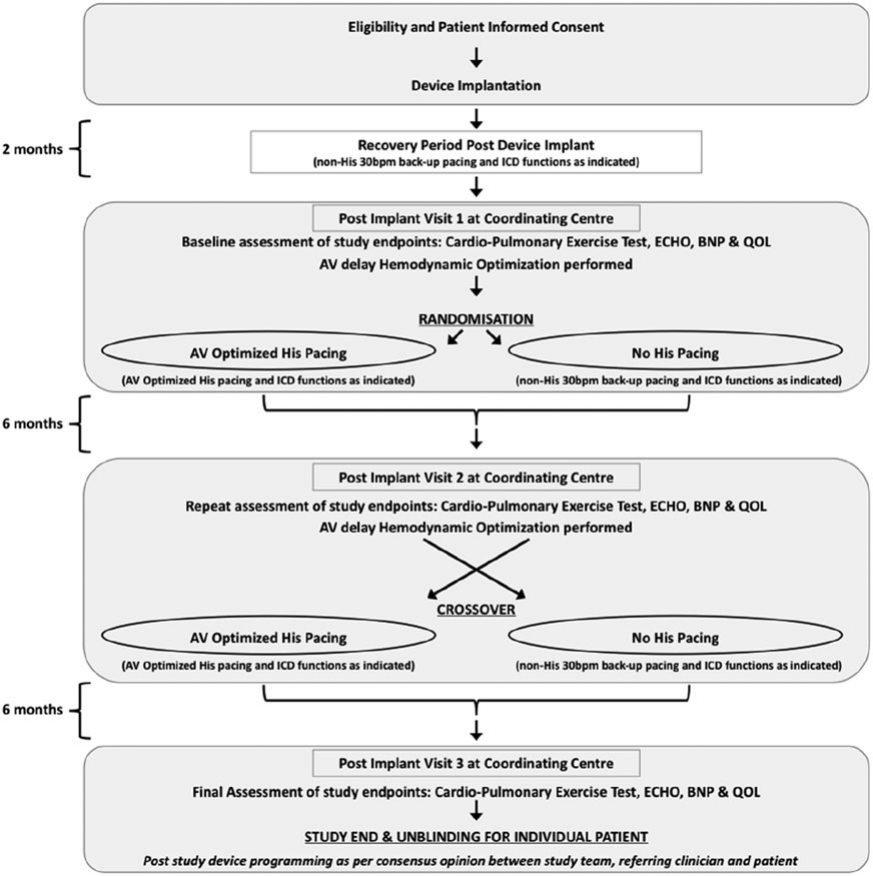
Patient study flow chart (AV, atrioventricular; BNP, B‐type natriuretic protein; ICD, implantable cardioverter defibrillator; QOL, quality of life).

A flow chart outlining the study design is provided in *Figure* *4*. In brief, after device implantation, patients are programmed to receive non‐His bundle backup pacing [VVI 30 b.p.m. (or DDI 30 b.p.m. if clinically indicated) utilizing either the right ventricular or coronary sinus lead if implanted], ICD therapies are enabled throughout the study in patients who have an ICD implanted. The 2 month run in period allows patients to recover from the implant procedure and ensures that the His lead has integrated into the tissue prior to randomization.

Two months after implantation, study participants visit the core centre to have baseline measurements recorded, which includes all endpoint measurements. AV delay optimization is performed using continuous non‐invasive blood pressure. Patients are then randomized to 6 months of AV optimized His pacing or 6 months of no His pacing. At the end of each 6 month period, all the endpoints are measured. Patients then enter the other arm of the study.

#### Randomization and allocation concealment

All patients have a commercially available CRT device implanted as part of the trial with a pacing lead permanently positioned on the His bundle. After the initial 2 month period, patients are randomized 1:1 to AV optimized His‐bundle pacing or no His pacing.

The study is double‐blinded with patients, and the staff conducting endpoint assessments both blinded to the pacing treatment allocation. To ensure blinding is maintained, special precautions are taken to prevent key staff from seeing the ECG even during exercise testing.

#### Maintaining blinding during the trial

Atrioventricular optimization is performed at each study visit by a member of staff blinded to treatment allocation. Before the optimization process, a separate non‐blinded staff member programmes the device to His pacing in readiness for the optimization. After the optimization, this non‐blinded study team member reprogrammes the device to either His pacing on or His pacing off according to the treatment arm, using the AV optimum determined by the blinded staff member during optimization.

During cardiopulmonary exercise testing, it is essential to monitor the ECG for patient safety. To ensure blinding is maintained, two members of the trial team are always present during a patient's test—one is blinded and the other not. The blinded member of the team leads the test but is not able to see the ECG. The role of the non‐blinded trial team member is to observe the ECG for arrhythmias that would necessitate the test be stopped for safety reasons. The unblinded member does not communicate with the patient or the blinded member unnecessarily.

Echocardiography is performed and analysed by a blinded member of staff. No ECG is recorded during the procedure. Pacing checks are only conducted by a designated unblinded investigator.

### Design of the atrioventricular delay haemodynamic optimization protocol

In this study, we use non‐invasive acute haemodynamic measurements to identify the optimal paced and sensed AV delays for individual patients.

In order to minimize the effect of noise, and so minimize the uncertainty of the optimization, we use the repeated alternation and parabolic fitting method. This approach has been shown to have a high level of reproducibility for determining the AV delay optimum. It has been described in detail previously and uses continuous non‐invasive beat‐by‐beat blood pressure using the Finapres Nova device (Finapres Medical Systems, The Netherlands).^32^ Briefly, continuous non‐invasive beat‐by‐beat blood pressure is recorded using the Finapres Nova device. Each tested AV delay is compared with a reference setting, and mean relative change in SBP is then calculated as the difference between the mean SBP at the reference setting (8 beats at the reference setting immediately before the transition) and the mean SBP at the tested setting (8 beats immediately after the transition to the tested AV delay). A minimum of six alternations are performed between the reference setting and atrial synchronous dual chamber His pacing and the mean of these alternations used to calculate the mean relative change for a particular programmed AV delay compared with the reference setting.

For sensed AV delay, the reference setting is sinus rhythm. For the atrial paced AV delay, the reference setting is AAI pacing at 10 b.p.m. above the patient's intrinsic rate.

**Figure 5:**
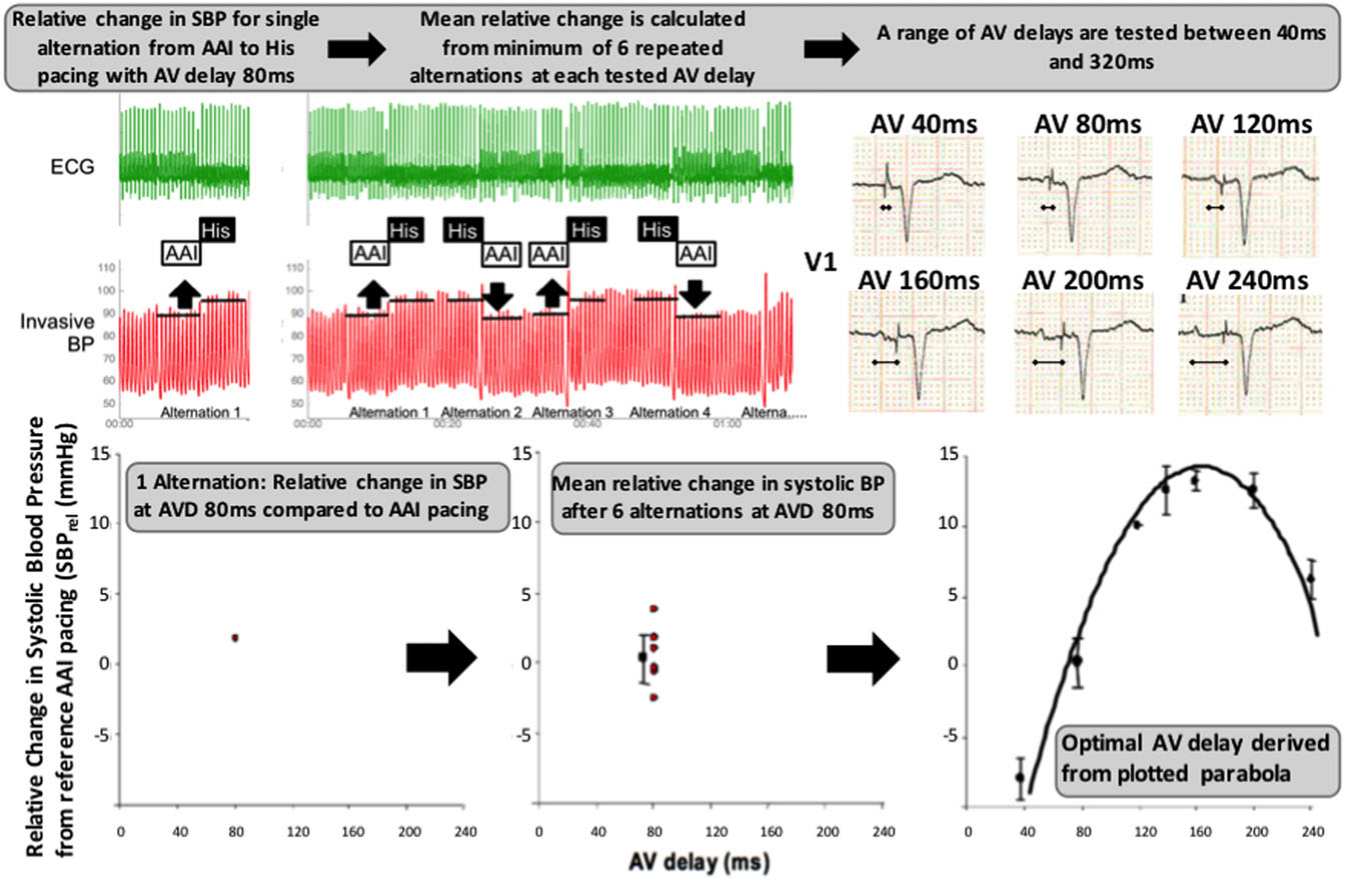
Atrioventricular (AV) delay optimization protocol. The relative change in systolic blood pressure (SBP) between 8 beats of AAI pacing and 8 beats of dual chamber atrial synchronous–His pacing at a given atrioventricular delay (AVD) is determined [AV delay = 80 ms in this example (top left)]. This is a single alternation (plotted bottom left). A minimum of six alternations are made for each AVD (middle sections top and bottom). This process is repeated for each AV delay between 40 and 320 ms (less if intrinsic AV conduction occurs before 320 ms). The optimal AV delay is then derived from the plotted parabola (bottom right). In this example, the optimal AV delay was 160 ms. BP, blood pressure; ECG, electrocardiogram.

The following programmed AV delays are tested: 40, 80, 120, 160, 200, 240, 280, and 320 ms, but the protocol is stopped if intrinsic ventricular activation occurs before 320 ms. *Figure* *5* demonstrates the AV optimization process.

#### Analysis plan

All study data are being recorded in our institution's clinical trial database (Inform, version 4.6). The trial is conducted through the Imperial College Clinical Trials Unit, which provides monitoring and trial oversight to minimize the amount of missing data.

The primary endpoint is peak oxygen uptake measured using cardiopulmonary exercise testing. The primary outcome will be analysed using a two‐level hierarchal model. We will include treatment, period, and sequence effect within the model. Random subject effects will also be included in the regression model if appropriate. Differences in secondary outcomes will also be analysed using a two‐level hierarchal model. Normality will be checked, and appropriate transformation or non‐parametric methods will be used if it is not met.

### Planned subgroup analysis

#### Right bundle branch block cohort

We expect one‐third of recruited patients to have RBBB. In these patients, His‐bundle pacing may shorten QRS duration by overcoming the RBBB (Supporting Information, *Figure* *S1*). Therefore, patients may theoretically obtain benefit from ventricular resynchronization in addition to that obtained from optimization of AV delay. Narrowing of QRS may occur by (i) positioning the pacing lead distal to the site of bundle branch block leading to an electrical bypass of the block; (ii) overcoming the block with sufficient stimulus to activate distal dormant tissue, relying on either source–sink relationships during pacing vs. intrinsic impulse propagation or the virtual electrode polarization effects or (iii) retrograde activation via capture of an upper septal branch of the His‐Purkinje system or (iv) in the case of non‐selective His‐bundle capture partial or full correction of the terminal RBBB pattern by fusion without recruiting the RBB fascicles.

We will perform a subgroup analysis to test for the possibility that RBBB patients show a larger benefit than the narrow QRS patients, which might signify the presence of dual benefit in bundle branch block patients.

#### PR interval

We will test whether there is a relationship between magnitude of improvement in the outcome measures with AV optimized His pacing and baseline PR interval. Such a relationship might be expected if there is a benefit from PR interval shortening. The PR interval of 200 ms or above was chosen as the entry for the HOPE‐HF because in the COMPANION trial, patients with a PR interval longer than 200 ms appeared to obtain greater benefit from CRT.^33^ However, the MADIT‐CRT study reported a larger difference in those whose PR interval was >230 ms^2^; we wanted to recruit a broad range of patients who might permit later evaluation of the effect of PR interval.

#### E‐A wave analysis

We will test whether the presence of E‐A wave fusion predicts the size of any His pacing benefit.

Baseline E‐A wave fusion on echocardiography suggests that there is inefficient left ventricular filling, and it is possible that it may be useful in selecting patients who are likely to benefit from AV optimization with His pacing.

#### Selective vs. non‐selective His capture

If there are significant numbers of patients with non‐selective His capture, we will perform an analysis to assess whether the type of His capture affects the outcome measures.

### Sample size calculation

The smoothed modified Bruce protocol in patients with heart failure has a test–retest variability of 2.4 mL/kg/min.^29^ Implantation of a CRT‐P in patients with broad QRS duration and heart failure results in a 0.5–2.5 (mean 1.5) mL/kg/min increase in peak oxygen uptake.^34^, ^35^ Acute haemodynamic data suggest that the benefit from His‐bundle pacing in long PR narrow QRS patients is around 60% of the effect seen with CRT in LBBB patients.^24^ We might therefore expect that the estimated increment in peak oxygen uptake of 60% would be 0.9 mL/kg/min. One hundred and twenty six patients would give 90% power at the 5% significance level to detect a 0.7 mL/kg/min change. To account for combined mortality and dropout rates as high as 21%, we will recruit 160 patients.

### Compliance and loss to follow‐up

It is anticipated that some patients may request a change in their treatment arm, particularly if there is a change in their symptom status. If this occurs, we will try to encourage patients to continue with their designated treatment until they cross over or complete the study as scheduled. If however the clinical team judges that it is necessary to turn on or off His pacing, this will be performed. Analysis will be on the standard intention to treat basis.

Follow‐up will be coordinated and carried out by a research team dedicated to this study. If patients decide they no longer wish to attend the core centre for follow‐up, then we will ask if they are willing at least to be contacted by the research team to ensure data on adverse events can be recorded.

### Steering committee

This committee is responsible for developing and monitoring the implementation of the protocol. The committee is supported by a statistician independent of the sponsor and is responsible for ensuring timely publication of the results.

### Data Safety Monitoring Board

The Data Safety Monitory Board (DSMB) is responsible for monitoring patient safety and will recommend that the trial is stopped prematurely if a significant increase in all‐cause mortality is noted in the active His pacing arm. The DSMB may request interim analyses should the safety data indicate that treatment is associated with important adverse events.

### Adverse events

All adverse events will be documented and reviewed by the study‐specific DSMB. Serious adverse events are defined as those which are not anticipated or not known to be related to the condition being studied or the intervention being used that would result in any of the following outcomes: death, life‐threatening condition, unexpected/unplanned inpatient hospitalization, and persistent or significant disability or incapacity.

### Timeline

Recruitment began in January 2016 and is expected to complete by the end of 2018. Follow‐up is therefore expected to end by approximately the end of 2019 and the trial to report in 2020.

## Discussion

If AV optimized His pacing is found to deliver clinical benefits to patients with heart failure and a long PR interval without LBBB, this procedure may become a new therapeutic option for this population of highly symptomatic patients. Pacing therapy for heart failure is not currently indicated for this population of patients; UK data suggest that they form approximately 20% of all heart failure patients.^5^

A positive result from this trial would provide randomized controlled evidence of physiological benefit of His‐bundle pacing in these patients.

## Conflict of interest

P.V. reports the following disclosures: speaker, consultant, research—Medtronic; consultant—Boston Scientific; consultant—Abbott. All other authors declare no conflict of interest.

## Funding

HOPE‐HF is an investigator‐initiated trial, funded by a British Heart Foundation (BHF) project grant (CS/15/3/31405). Medtronic are supporting the study, by supplying the His pacing leads, sheaths, CRT‐P devices (in those patients who do not have a clinical indication for ICD implantation) and the additional cost of a CRT device over dual chamber ICD in those indicated for ICD implantation. Z.W. is supported by the BHF (FS/13/44/30291), D.K. and M.S.S. are BHF Clinical Research Training Fellows (FS/15/53/31615 and FS/14/27/30752), respectively. D.F. holds a BHF Fellowship FS/10/038.

## Supporting information


**Figure S1.** (A) Normal ventricular activation, with activation travelling from the AV node down the Bundle of His and then into the Left and Right Bundle Branches. This illustration demonstrates that fibres within the bundle of His are already predestined for their respective Bundle Branches. (B) Proximal site of RBBB, (C) a pacing lead has been positioned in a site distal to this block allowing an electrical bypass of the RBBB and thus reversing the electrical abnormality. (D) Distal site of RBBB (unfeasible for pacing lead to be positioned distal to this). (E) Remote electrical activation with a proximally placed lead could reverse this electrical abnormality. This could occur (1) because of high pacing outputs, (2) as a consequence of the source‐sink‐theory and a high likelihood of their being diseased fibres already in the proximal location or (3) the virtual electrode polarization theory. (F) Highlights that each bundle branch has multiple branches. (G) A high septal branch may fortuitously be activated with a conventionally placed His lead. This could result in retrograde activation down the branch back to a place in the bundle branch which is distal to the site of block allowing antegrade activation from this site forward.
**Table S1.** Summary characteristics of selective and non‐selective his bundle pacing.
