# Supplementary material for: Rationale and design of the randomized multicentre His Optimized Pacing Evaluated for Heart Failure (HOPE‐HF) trial
Source: ESC Heart Fail. 2018 Jul 9;5(5):965–76. doi: 10.1002/ehf2.12315 (PMC6165934; doi:10.1002/ehf2.12315)
Supplement: Supplementary file 1 — Figure S1. (A) Normal ventricular activation, with activation travelling from the AV node down the Bundle of His and then into the Left and Right Bundle Branches. This illustration demonstrates that fibres within the bundle of His are already predestined for their respective Bundle Branches. (B) Proximal site of RBBB, (C) a pacing lead has been positioned in a site distal to this block allowing an electrical bypass of the RBBB and thus reversing the electrical abnormality. (D) Distal site of RBBB (unfeasible for pacing lead to be positioned distal to this). (E) Remote electrical activation with a proximally placed lead could reverse this electrical abnormality. This could occur (1) because of high pacing outputs, (2) as a consequence of the source‐sink‐theory and a high likelihood of their being diseased fibres already in the proximal location or (3) the virtual electrode polarization theory. (F) Highlights that each bundle branch has multiple branches. (G) A high septal branch may fortuitously be activated with a conventionally placed His lead. This could result in retrograde activation down the branch back to a place in the bundle branch which is distal to the site of block allowing antegrade activation from this site forward. Table S1. Summary characteristics of selective and non‐selective his bundle pacing. [file EHF2-5-965-s001.docx]

Figure S1 (Supplementary material)


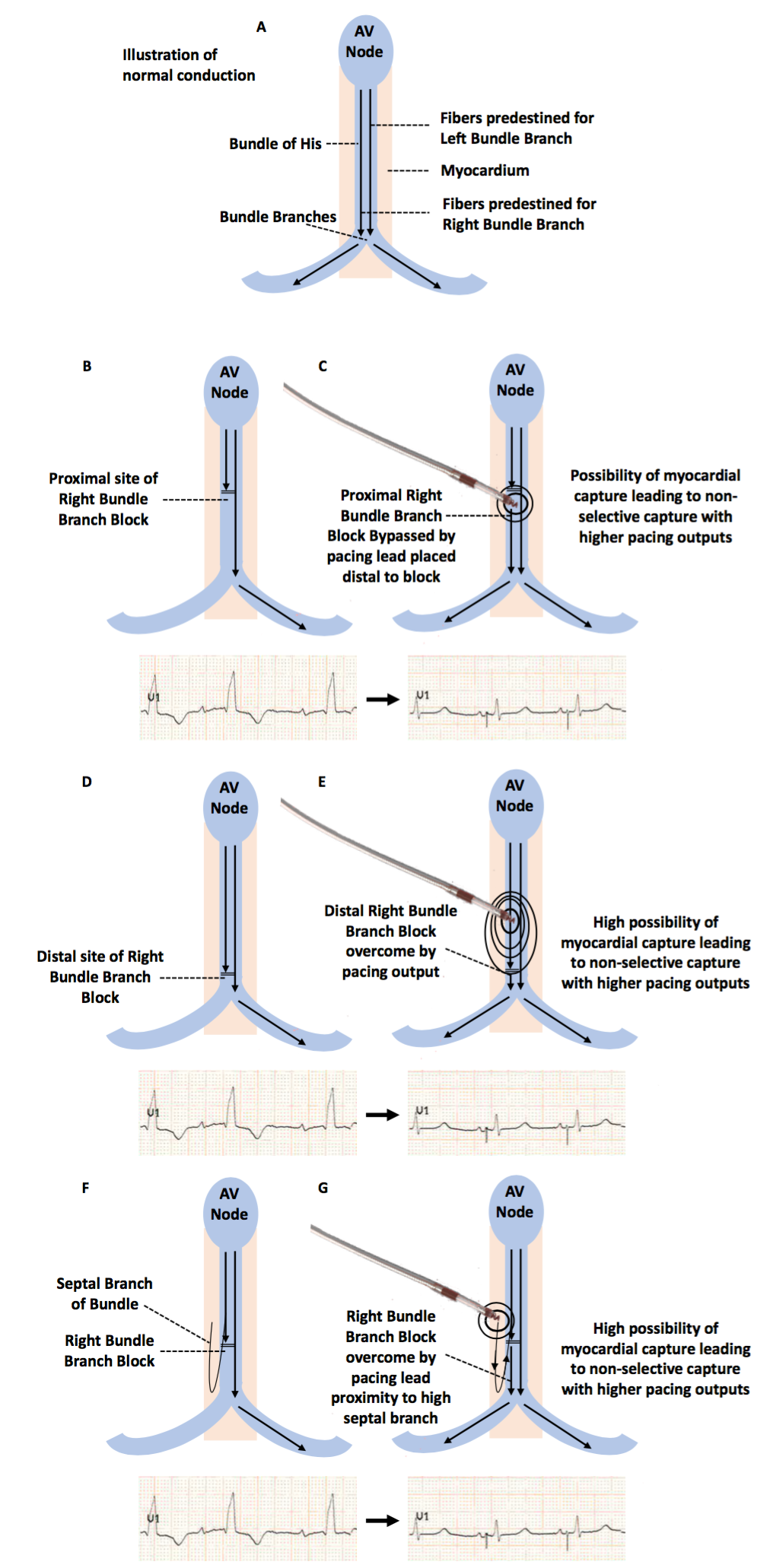


**Figure S1**

**A,** Normal ventricular activation, with activation travelling from the AV node down the Bundle of His and then into the Left and Right Bundle Branches. This illustration demonstrates that fibres within the bundle of His are already predestined for their respective Bundle Branches.

**B,** Proximal site of RBBB, **C,** a pacing lead has been positioned in a site distal to this block allowing an electrical bypass of the RBBB and thus reversing the electrical abnormality.

**D,** Distal site of RBBB (unfeasible for pacing lead to be positioned distal to this) **E,** Remote electrical activation with a proximally placed lead could reverse this electrical abnormality. This could occur (1) because of high pacing outputs, (2) as a consequence of the source-sink-theory and a high likelihood of their being diseased fibres already in the proximal location or (3) the virtual electrode polarization theory.

**F,** Highlights that each bundle branch has multiple branches, **G,** A high septal branch may fortuitously be activated with a conventionally placed His lead. This could result in retrograde activation down the branch back to a place in the bundle branch which is distal to the site of block allowing antegrade activation from this site forward.

Table S1 (supplementary material)

| Evidence for Selective His Bundle capture only (without localized RV capture) |
| --- |
| - The pacing stimulus is followed by an isoelectric interval before the onset of the QRS - The isoelectric interval from pacing stimulus to QRS onset is equal to the interval from the native His electrogram to QRS onset (HV interval). [If the H-V interval is prolonged there may be shortening of the Stim-QRS interval but will still be greater than 30ms.] - The stim to end QRS interval is equal to or shorter than the native His electrogram to end of QRS interval. [Shortening may occur due to resolution of bundle branch block or shortening of a prolonged HV interval.] - The paced QRS morphology is identical or virtually identical to the native QRS morphology - A discrete local ventriculogram is present on the pacing lead separate to the pacing stimulus |
| Evidence for Non-Selective His Bundle capture (local RV capture in addition to His Bundle capture) |
| - The interval from pacing stimulus to QRS onset is very short and maybe zero as there is no isoelectric interval between the stimulus and the QRS - The paced QRS duration will usually be longer than the native QRS duration (due to the presence of the pseudo-delta wave representing antero-septal RV myocardial capture occurring prior to His-Purkinje mediated ventricular activation) however the duration from native His electrogram to the end of the QRS is equal to the duration from pacing stimulus to the end of the QRS. [Shortening may occur due to resolution of bundle branch block or shortening of a prolonged HV time] - The overall electrical axis of the paced QRS will be concordant with the electrical axis of the intrinsic QRS - The local discrete ventriculogram on the His pacing lead will be largely absent due to instantaneous ventricular capture |

**Table S1** Summary Characteristics of Selective and Non-Selective His Bundle Pacing
